# Supplementary material for: Exploring individual variation in associative learning abilities through an operant conditioning task in wild baboons
Source: PLoS One. 2020 Apr 6;15(4):e0230810. doi: 10.1371/journal.pone.0230810 (PMC7135308; doi:10.1371/journal.pone.0230810)
Supplement: S1 Appendix — (DOCX) [file pone.0230810.s001.docx]

**Appendix S1**

Demographics of all identifiable individuals in both study troops

| Sex | Troop | Adult | Juvenile | Total |
| --- | --- | --- | --- | --- |
| Male | *J* | 7 | 14 | **21** |
|  | *L* | 5 | 19 | **24** |
|  | *Total* | **12** | **33** | **45** |
| Female | *J* | 18 | 5 | **23** |
|  | *L* | 18 | 1 | **19** |
|  | *Total* | **36** | **6** | **42** |
