# Supplementary material for: Exploring individual variation in associative learning abilities through an operant conditioning task in wild baboons
Source: PLoS One. 2020 Apr 6;15(4):e0230810. doi: 10.1371/journal.pone.0230810 (PMC7135308; doi:10.1371/journal.pone.0230810)
Supplement: S2 Appendix — Shown are the total numbers of baboons tested of each age-sex class in each troop, including the total. The percentage of the population that the sample represents is presented in brackets. (DOCX) [file pone.0230810.s002.docx]

**Appendix S2**

The numbers of baboons tested according to sex, age and troop, and the total number and (in brackets) percentage of the population the sample represents

| Sex | Troop | Adult | Juvenile | Total |
| --- | --- | --- | --- | --- |
| Male | *J* | 4 | 7 | **11 (52%)** |
|  | *L* | 2 | 7 | **9 (38%)** |
|  | *Total* | **6 (50%)** | **14 (40%)** | **20 (42%)** |
| Female | *J* | 7 | 3 | **10 (43%)** |
|  | *L* | 7 | 1 | **8 (42%)** |
|  | *Total* | **14 (38%)** | **4 (66%)** | **18 (43%)** |
