# Supplementary material for: Exploring individual variation in associative learning abilities through an operant conditioning task in wild baboons
Source: PLoS One. 2020 Apr 6;15(4):e0230810. doi: 10.1371/journal.pone.0230810 (PMC7135308; doi:10.1371/journal.pone.0230810)
Supplement: S3 Appendix — Shown are the total numbers of baboons tested of each dominance rank and neophilia level, including the total. The percentage of the population that the sample represents is presented in brackets. For the purposes of this table, dominance ranks were grouped evenly into categories of “low-rank”, “medium-rank” and “high-rank” according to tertiles; while neophilia levels was grouped evenly into categories of “low-neophilia”, “medium-neophilia” and “high-neophilia” according to tertiles. (DOCX) [file pone.0230810.s003.docx]

**Appendix S3**

The number of baboons tested according to their dominance rank (low, medium or high) and neophilia level (low, medium or high) and the total number and (in brackets) percentage of the population the sample represents

| Trait | Level | Total |
| --- | --- | --- |
| Dominance Rank | *Low* | **10 (11%)** |
|  | *Medium* | **18 (20%)** |
|  | *High* | **10 (11%)** |
|  | *Total* | **38 (42%)** |
| Neophilia | *Low* | **7 (8%)** |
|  | *Medium* | **22 (25%)** |
|  | *High* | **9 (10%)** |
|  | *Total* | **38 (42%)** |
