# Supplementary material for: Exploring individual variation in associative learning abilities through an operant conditioning task in wild baboons
Source: PLoS One. 2020 Apr 6;15(4):e0230810. doi: 10.1371/journal.pone.0230810 (PMC7135308; doi:10.1371/journal.pone.0230810)
Supplement: S4 Appendix — Shown are the Spearman correlation coefficients of the predictor variables used in the GLMM models. Sample size is N = 38 individuals in all cases. Individual vigilance and total time were calculated as the median across all trials (1–5). First choice refers to the first choice between each pile of corn in the first trial (Palatable, P; Unpalatable, U). (DOCX) [file pone.0230810.s004.docx]

**Appendix S4**

Shown are the Spearman correlations coefficients of the predictor variables used in the GLMM models. Sample size is N=38 individuals in all cases. Individual vigilance and total time were calculated as the median across all trials (1-5). First choice refers to the first choice between each pile of corn in the first trial (Palatable: P, Unpalatable: U).

|  | Sex | Age | Rank | Neophilia | Vigilance | Troop | Fst.Ch | Total Time |
| --- | --- | --- | --- | --- | --- | --- | --- | --- |
| Sex | 1.00 |  |  |  |  |  |  |  |
| Age | 0.54 | 1.00 |  |  |  |  |  |  |
| Rank | 0.59 | -0.01 | 1.00 |  |  |  |  |  |
| Neophilia | 0.12 | 0.50 | -0.20 | 1.00 |  |  |  |  |
| Vigilance | 0.19 | -0.32 | 0.49 | -0.18 | 1.00 |  |  |  |
| Troop | 0.01 | 0.04 | 0.08 | 0.15 | -0.06 | 1.00 |  |  |
| First Choice | 0.38 | 0.20 | 0.16 | 0.11 | 0.08 | 0.09 | 1.00 |  |
| Total Time | 0.14 | -0.41 | 0.49 | -0.09 | 0.63 | -0.16 | 0.09 | 1.00 |
