# Supplementary material for: Exploring individual variation in associative learning abilities through an operant conditioning task in wild baboons
Source: PLoS One. 2020 Apr 6;15(4):e0230810. doi: 10.1371/journal.pone.0230810 (PMC7135308; doi:10.1371/journal.pone.0230810)
Supplement: Attachment — Submitted filename: Reviewer comments_FINAL.docx [file pone.0230810.s005.docx]

**Response to reviewers**

**We would like to thank the comments of the Academic Editor and two anonymous reviewers for their helpful comments and suggestions. Our responses below are presented on a point-to-point basis in bold. When relevant, we include the line numbers that correspond to changes made in the manuscript version without track changes.**

**Reviewer 1**

General comments:

This is an interesting study on individual determinants of learning performance in free-ranging baboons. There are still few datasets on free-ranging animals’ cognition, and it seems to me that these tests were conducted in a careful manner, for instance the selection of focal individuals ensure representativity of the different categories of individuals, regular inter-trial intervals, and in avoiding social learning (although see my question below about how the kernels that were eaten when an individual interrupted another individual’s trial were treated).

1. However I have serious reservations about the statistical approach, for several reasons (see below for more details, but here are the main two): (i) 40% of the dataset was thrown away due to individuals first selecting the unpalatable kernels; this could have instead been controlled for statistically, and (ii) the Y variable also threw away potentially useful biological information by transforming a proportion correct per trial into an arbitrary binary 0-1 variable (the raw proportion could have been implemented as a binomial GLMM with the cbind function in lme4 in R).

**We thank you for highlighting these points. Based on these suggestions, we have: (1) controlled for the first choice individuals made in the first trial by including a binary variable (P = palatable; U = unpalatable) as a covariate in the model; (2) included a new analysis that evaluates each “kernel choice” as a response variable, which we coded as a binary variable (1 = Correct; 0 = Incorrect) to analyse whether baboons learnt the association in each trial (1-5) independent of each other trial; and (3) used the proportion of correct kernels using the “cbind” function to analyse learning across trials.**

2. At the moment, given the authors cannot statistically demonstrate a positive and significant effect of trial number, the conclusions drawn from these analyses cannot be interpreted in the light of the hypotheses, i.e. individual sources of variation in learning performance. The fixed effects that revealed significant as main terms only suggest that some categories of individuals were more successful overall, not that they changed their preference at a higher rate (which would have been shown by significant interactions with trial number). The latter is necessary in a protocol like this one, where each individual was only tested on 1 correct stimulus, so as to rule out individual-level preferences and other non-learning explanations that can be causing the main term effect.
**Our new analyses demonstrate that baboons do not learn the association across trials (i.e. there is no interaction with trial number, as the reviewer highlights)**

3. Given the small N, an alternative modelling approach could be to model *all choices* in a binary GLMM where Y is unpalatable=0 & palatable=1, and including ‘’kernel number’’ (within each trial, 1-10) and ‘’trial number’’ (2-5) as fixed terms. This may potentially reveal some learning within trials even if you cannot evidence it between trials (when memory may have faded away 3 days later), and may be a way to examine your untested explanation of the results that ‘’rather than gradually improving their performance over several trials, all animals learnt the intended association rapidly’’. You could then test for interactions of your fixed terms with kernel number IF this term is significant, but be aware that fixed-term interactions need to be tested while controlling for potential individual differences in learning rate (i.e. need to include a random slope effect, cf. Schielzeth & Forstmeier 2010 Behav Ecol 20, 416-20).
**As mentioned above, following the reviewer’s suggestion, we now include a kernel-level analysis to evaluate the support for three different hypotheses: (1) Individuals learnt the association rapidly in the first trial. For this analysis we used all the choices individuals could have made within the first trial. (2) Individuals learnt the association in trials 2-5 independently in each trial i.e. there was within-trial learning. To test this hypothesis, we repeated the first analysis for each subsequent trial, using all the kernel choices individuals had in a given trial. (3) Individuals learnt the association gradually, across trials (i.e. across-trial learning). To test this hypothesis, we used the proportion of correct kernels within the first 20 choices with the “cbind” function in R and included Trial number as a fixed effect. We decided this approach used the data in the most optimal way and could detect patterns that we had previously missed when analysing only the proportion of the first ten kernels in each trial. In all sets of analyses we tested for interactions with the fixed effects; however, we were unable to fit a random slope as the data used lacked sufficient power to fit an additional random effect.**

Detailed comments:

1. L34 : ‘phenotype’ is vague, specify which aspects of the phenotype

**We have now included examples of what we refer to by “phenotype” (re-worded here to “phenotypic traits”: L33) and state (L33-34)**

2. L34-6: it doesn’t make sense to claim animals were learning quickly but did not end up preferring the best food. This will need to be adjusted after re-analysis.
**The abstract has been re-adjusted to reflect the new results and discussion.**

3. L40: why wouldn’t this be important for dominants too?

**The abstract has been re-worded following the new analyses.**

4. L51-2 + throughout: check/update order of references
**Done.**

5. L55: Reference 3 seems ill-chosen here as this is a study on intraspecific, no interspecific variation

**This reference has been removed.**

6. L67-70: this is an interesting idea, which doesn’t seem to come across very clearly at the moment. For instance, is 2500 trials much larger than what a juvenile crow or chimpanzee may go through in the wild before being proficient tool-users? What is meant exactly by ‘may obscure the adaptive significance of cognitive processes’?
**The point we wanted to make here is that captivity allows for extensive training and testing, exposing animals to stimuli at a rate that may not be possible in wild conditions, particularly with regards to tasks that are not ecologically relevant for the species (as is the case for this reference). While it is likely wild animals do have multiple attempts until they are proficient tool-users, there is little or no evidence as to the time-frame in which to develop these abilities. We have now re-worded the sentence to “As such, conclusions obtained from captive studies may not be entirely representative of natural processes, particularly because opportunities to develop a given ability may be quite different for captive and wild animals” to clarify our rationale (L66-69).**

7. L80: any reference available to support this? Also, does this behaviour leads to avoiding distasteful fruits? If so it would be nice to underline this as it supports more strongly the ecological relevance of your test.

**We have now included a few sentences (and relevant references) that address this comment (L79-81).**

8. L87: sources

**As this sentence is an introduction for the hypotheses which were tested in this manuscript, which are described in detail in the following paragraphs, we did not list all the sources at this point**.

9. L122: Ref 49 didn’t include cognitive performance assessment if I’m not mistaken

**This references has now been removed.**

10. L147-155: if these behaviours and methods have been published before please insert citation here

**We have now included a reference (i.e. 1: L170) for the methods used to obtained the dominance hierarchies.**

11. L156: In Greenberg’s model (and in your previous section), neophobia and neophilia are two distinct axes. It would thus be ideal to identify to which of the two your test belongs and label it as such. If you can do so, then there is no point in using the vague term ‘boldness’, which doesn’t indicate this is risk-taking relative to novelty (and not relative to predation, etc.). It seemed to me this was one of the recommendations in Carter et al 2013?

**Thank you for pointing this out. We have now changed all mention of boldness or personality to “neophilia” for consistency. Furthermore, we have clarified that neophilia and neophobia are two separate responses as was indeed suggested by Greenberg [2]. However, we would like to clarify that our use of the term “boldness” indicated risk-taking relative to novel food items such as the ones presented here and described in Carter et al., 2013 [3].**

12. L159: please report repeatability estimate from the original paper here, as this is a crucial piece of information.

**We have now included the repeatability estimate as requested (L174).**

13. L161-2: ratios do not control perfectly for their denominators, and thus there may be some effect of individual differences in trial duration when examining vigilance rate as a predictor (to examine this, look at the relationship between your ratios and trial duration, there may still remain a weak correlation). This is one reason often given to use alternative approaches, such as including trial duration in the model, or using a residual value.

**We have now included the total trial time as an additional fixed effect and examined vigilance instances as a whole number rather than a ratio (L333-337).**

14. L163-4: why not in the current trial? This may become clearer when I keep reading but for now doesn’t seem justified

**Our rationale here was that the effect of being vigilant in a given trial would affect individuals’ performance in the next trial (i.e. learning across trials) as learning and vigilance behaviour cannot happen at the same time [e.g. 4]. This has been clarified at L333-335.**

15. L167: can baboons be colour-blind for this specific colour discrimination, like humans? This would be an important point to make in the discussion if they are. It could also potentially reduce performance in males, if this was the case.

**Compared with New World Monkeys which are dichromats, all Old World Monkeys are uniformly trichromats [5]. Although this doesn’t exclude the possibility of inherited colour-blindness in baboons, as far as we know there is currently no evidence that this is the case. However, even if some male baboons are colour-blind, they may have still been able to learn the differences between the piles of corn based on the different hues they experience.**

16. L196: not sure what time is sunrise at that location but 6h-10h seems like a potentially big difference in hunger (i.e. empty stomach vs after morning foraging). Can you please insert information that time of day for the tests didn’t differ significantly between your individual categories, or didn’t affect learning performance? Or maybe this time period is typical for primates, and could be supported with a reference.

**Baboons show higher levels of foraging during morning periods compared to the afternoon, when they are likely more satiated [see: 6]. The purpose of testing animals in the early morning was to present food items during a period when baboons would be more motivated to approach the task. Since all of our tests subjects approached the task immediately after seeing it, we believe this is evidence that there was a similar level of motivation across the sample.**

17. L205-6: this seems like an arbitrary response variable that is throwing away important variation. The appropriate response here is a proportion in a binomial GLMM. In R you can build Y from: cbind (nb of successes, nb of failures). This will still allow including the individuals with a different number of trials.
**See our response to comment #3 above.**

18. L213-5: isn’t there a risk that this procedure inflate type I error? Why not use model selection instead? This may not be problematic but given discussions about the validity of stepwise procedures in the last 10 years, I feel this choice needs to be justified.
**At the risk of overparametrization in our models, we decided to use stepwise elimination.**

19. L218: how were these events integrated in the data, i.e. if some dominant individuals interrupt some other baboon’s trial and eat some corn, they would have had more information than those not doing so. How was this taken into account? If it was rare enough that it seemed not necessary to do so please indicate explicitly.
**We specified at L232-233 that individuals who interrupted a trial were not tested because they might have learnt the association and further have elaborated on this at L233-235.**

20. L223-4: this seems a shame given all the hard work that went into the field trials (=loss of 15 individuals/38=40%!). This also risks biasing your nicely-balanced representation of sex, age classes, etc. described on L168-170. Instead this should be controlled for statistically (adding a fixed term for first choice ‘palatable’ or ‘not’), or you should demonstrate statistically that these individuals’ datasets cannot be used as they didn’t learn.

**Thank you for this comment. In spite of our best efforts to include the full sample, we ultimately decided to subset the sample when analysing within-trial learning (i.e. Trial 1 and Trials 2-5 separately). This is because the baboons ate each pile of kernels entirely (which we now term “bulk feeding” in the manuscript) rather than switching between both piles throughout a trial. As a result, the baboons who bulk fed did not have an opportunity to learn as they did not experience both options. The subset we now use includes only those individuals that experienced both options before consuming a pile of kernels entirely. However, following the reviewer’s suggestion, we used the full sample in our across-trial analyses, including a fixed effect of “first choice” to control for this.**

21. L234-5: I don’t understand this here; you cannot change the reference value for Trial number (order matters so it should remain the first trial that is included in the dataset, i.e. 2); this is not categorical but discrete. You may have needed to do this if you had e.g. 3 age categories, but age and sex are dichotomous, while rank and personality are continuous...

**This has now been removed.**

22. L250-1: but this doesn’t speak against learning per se as you have few trials and animals make take some time to learn. The effect of trial number as a fixed predictor will be more revealing on this.
**We have removed this line, as well as used trial number as a fixed effect.**

23. L251-2: to me this is useful to show that the bitter taste was effective, but will not be effective to demonstrate learning, as your modelling approach suggests you are mostly interested in between-trials data.
**We have updated our statistical analysis to evaluate within-trial learning as well.**

24. L344-5: you have these data, so you should test this instead of relying on an untested explanation, i.e. by including trial 1 in the dataset and see if trial number is significant, or by analysing choices within trial 1.
**We have now analysed kernel choice within the first trial as suggested. We made two separate models to evaluate learning in this trial: (1) one, evaluating learning based on the order in which the correct kernels were consumed (1-40); (2) another, evaluating whether the order of consumption in this trial was determined by individuals’ phenotype/state.**

25. L422: Typo in the journal name? I didn’t check this section in details but it seems like latin names have not been italicised, some journal names are full and others abbreviated, etc. Please check the journal policy and adjust.

**These errors were part of the software we are using. We have now gone through the Reference section and edited these errors as suggested.**

Figures: we need to see the data plotted over trial numbers to visually assess learning.

**We have now included an Appendix (Appendix 4) that shows the data plotted over trials as suggested. The plot also includes the relationship between trial and kernel number.**

**Reviewer 2**

General Comments:

In this manuscript the authors measure the learning abilities of wild baboons by assessing the ability of individuals to correctly associate colour with a palatable food source (versus a distasteful one). Learning assessments were analysed in conjunction with the sex, age, and social ranking of each individual and their propensity to investigate novelty, in order to determine whether differences in learning are correlated to an individual’s status or phenotype. In general I found this manuscript to be interesting and well written. More studies on cognition in wild populations are critical if we are to better understand the evolutionary causes and consequences of intra-specific variation in learning abilities. However, as these authors have discovered, studies with wild organisms can be quite a challenge! I believe there are a number of issues that need to be addressed, these are outlined below:

Major comments:

1. My main concerns are related to the analysis and interpretation of results. Throughout the Results and Discussion sections the author suggests that there is little evidence of the baboons learning the set task, except for the lower ranking/ shy animals which learnt rapidly in the first trial. There isn’t support for either of these interpretations in the current presentation of results. It may be that there was no strong preference for the non-bitter kernel or the task was too simple (in which cases the associative task was not ideal for assessing learning), but again there are currently no supportive results. There has been much information loss due to the grouping of individuals choices (arbitrary it seems) as a binomial variable. Can the choice and consumed data be plotted as a proportion of correct choices for each trial (e.g. Jaumann, Scudelari & Naug et al. 2013)? This way learning can be assessed both within and between trials.

**In response to Reviewer 1’s comments, we have changed our statistical analysis to avoid this issue (please see our response to comment #3 and #20 of Reviewer 1).**

2. Why were only the first 10 kernels in each trial included in the assessment? The number of kernels provided and the number consumed by each individual in each trial is important information as their experience (and perhaps also how hungry they were) with the association and will vary if a different number of kernels are consumed in each trial or between trials (some individuals had more trials). This should be accounted for in analysis.

**We thank the reviewer for this comment. We have now expanded the dataset to include all the kernel choices made in every trial for our within-trial analysis and the proportion of correct kernels within the first 20 kernels for our across trial analysis. The latter amount corresponds to the number of kernels available in each pile of corn. While it is indeed possible that animals differed in their level of hunger, we attempted to control for this by testing individuals during the morning foraging period (please see our response to Review 1, comment #16 above). The relatively low number of kernels left and the few instances in which this happened further supports the idea that all animals had a similar level of motivation to consume a high-value resource.**

3. Age and vigilance are recorded but no results are reported. Please clearly indicate how these traits were analysed (included in GLMM?) and what the results were.

**Age was included in all models testing the interaction of “Kernel number” (i.e. M_T1*_) or “Trial number” (i.e. M_T2-5*_) and individuals’ phenotypes/states; while vigilance was only included in the latter model.**

4. I would like to see the inclusion of a table that shows the sample size breakdown for the different social ranks and degrees of boldness and any correlations that exist between any of the traits/states measured.

**A table with the sample size breakdown based on social rank and the degree of neophilia (changed from boldness for consistency) has now been provided (Appendix 2, Table B). Any potential correlations were controlled for during the testing of multicollinearity (L284-286).**

5. Did models test for interactions between variables? It seems reasonable to expect that some of the variable measured will have an interactive relationship, the effect of these interactions on task performance should be assessed.

**For the purposes of this study and the evaluation of the hypotheses, we did not test any interactions between the five core variables as we did not have sufficient statistical power with this dataset and it was not part of our original predictions to test.**

6. Introduction Line 54: I suggest some rewording/reordering of ideas in this sentence to improve clarity. Currently the proximate and ultimate explanations are mixed and the role of the costs associated with learning needs to be mentioned. See below for an example. I have also provided some additional references that would be good to included.
Ultimately learning enables populations to adapt to their environmental (3,12), but individuals within a population may exhibit genetic differences in learning abilities (13,14) due to the costs associated with learning (Foley & Lee 1991; Mery & Kawecki 2003; Dunlap & Stephens 2016; Evans et al. 2017), and the different environments experienced (different environments may select for different learning capacities).
• Foley RA, Lee PC. Ecology and energetics of encephalization in hominid Evolution. Philosophical Transactions of the Royal Society of London Series B-Biological Sciences. 1991; 334, 223-232.
• Mery F, Kawecki TJ. A fitness cost of learning ability in Drosophila melanogaster. Proceedings of the Royal Society B-Biological Sciences. 2003; 270, 2465-2469.
• Dunlap AS, Stephens DW. Reliability, uncertainty, and costs in the evolution of animal learning. Current Opinion in Behavioral Sciences. 2016; 12, 73-79.
• Evans LJ, Smith KE, Raine NE 2017. Fast learning in free-foraging bumble bees is negatively correlated with lifetime resource collection. Scientific Reports. 2017; 7, 496.

**We indeed mention in L49-51 the costs and benefits associated with learning; however, our intention here was to mention how cues can be associated with costly outcomes, for instance, in cases where colouration signals inedibility.**

7. Introduction Line 126: Need to be consistent when referring to this phenotypic trait, you have used the terms personality, neophilia/ neophobic, and boldness/shyness. Decide on one of these combinations, define in this section of manuscript and then use throughout.

**We now use the term “neophilia” throughout the manuscript for consistency. We define it in L138-139 and describe how it was measured in L171-175.**

8. Experimental procedure Line 166: Why were the colours green and red chosen? Do baboons have any innate colour preferences and/or visual limitations? Could individual differences in these traits have biased the results?
**Please see our response to comment #6 and #15 raised by Reviewer 1. Additionally, we attempted to control for any possible innate differences in colour preferences by presenting the reverse association to each troop (i.e. in J troop the palatable kernels were red, while palatable kernels were green in L troop) and we included Troop membership as a fixed-effect in our analyses.**

9. Experimental procedure Line 176: Does the bitter solution have an odour that could be used by baboons when deciding with kernel to consume?

**Since the liquid used to make the kernels bitter had a distinct smell, we had likewise assumed that odour could have been used as a cue to differentiate between both piles of corn. We recorded the number of sniffing instances each baboon had in each trial; however, our preliminary analyses did not reveal sniffing to have an effect on which pile of kernels the baboons chose to eat first. Moreover, sniffing was not observed in all trials, even for those individuals that sniffed the kernels when they were first presented. We now include a few sentences to address this in L413-415 of the “Results” section.**

10. Discussion Line 290: These two statements are contradictory – “there was no consistent effect of any phenotype/state and trial interaction” and “we did find that lower ranking and shyer animals were consistently better in task performance”

**We have deleted these statements from the updated discussion.**

11. Discussion Line 348: Provide references for your mention of “other studies of wild animals that failed to identify individual differences in learning”. It would be good to discuss the importance of using the right task to assess learning somewhere in your discussion – this paragraph may be a good place.

**We have modified this line and as suggested, we have now included a brief paragraph in the Discussion section that touches on the importance of using the correct tasks to measure cognition in wild animals (L539-556).**

12. Discussion Line 367: I’m not convinced by the summary paragraph… your key finding appears (if it can be shown in the results as per comments above) to be that status and the willingness of individuals to engage with novelty affect task performance, so mention this first. The challenges working with wild populations is not just how to capture the variation in cognitive ability among individuals but how to interpreting this variation. In most cases it is impossible to know the experiences of wild individuals prior to capture/testing and how such experience might differ among individuals. Here you use a valid approach to try to address this caveat by collecting additional information about your study subjects and taking into account their effect on task performance. Good to point out to readers that you understand the challenges involved in answering these types of questions with wild animals and have devised an approach to try and address some of these challenges. Secondly, your results demonstrate the need to designing a task simple enough to be used by individuals in the wild, yet sufficiently robust to reveal variation in a focal cognitive trait.
**We agree that one of the challenges of testing animals, particularly those in wild conditions, is identifying external factors that affect behaviour; we are glad the reviewer recognized the efforts made to control for such sources of variation. Likewise, we appreciate that the reviewer addressed the difficulty in finding suitable tasks to test wild animals. We have now changed this paragraph to take into consideration the reviewer’s suggestions.**

Minor comments:

1. Introduction Line 68: I am not quite sure what you mean by ‘obscure the adaptive significance’
**Please see our response to comment #6 raised by Reviewer 1 (L66-69).**

2. Methods Line 228: In brackets list the specific phenotypes and states looked at and remove ‘except in the case of vigilance, for which no such learning is predicted’

**We specify the traits we refer to in this sentence beforehand (L298-299). We have removed the suggested sentence from the manuscript.**

3. Results Line 247: change text to ‘Together, these individuals were tested over 102 trials (mean number of trials per individual =’
**These sentence has been changed as no longer forms part of the manuscript; however, we took into account the reviewer’s suggestion and included the mean number of trials per individual in the “Results” section (L400-401).**

4. Results Line 247: In methods section the authors state that individuals undertaking less than 3 or 4 trials were not included in analysis, if this is the case shouldn’t the range of tests per individual be 3 -5?
**Done (L401).**

5. Figure 1 Line 264: In supporting text for both figures please provide stats indicating significance.
**We have included statistical values in all figures as suggested.**

**REFERENCES**

1.	Netto WJ, Hanegraaf PLH, DE VRIES H. Matman: A Program For The Analysis Of Sociometric Matrices And Behavioural Transition Matrices. Behaviour [Internet]. 2008 [cited 2018 Jun 14];125(3–4):157–75.

2.	Greenberg R. The Role Of Neophobia And Neophilia In The Development Of Innovative Behaviour Of Birds. In: Animal Innovation [Internet]. Oxford University Press; 2003 [cited 2016 Dec 2]. p. 175–96.

3.	Carter AJ, Marshall HH, Heinsohn R, Cowlishaw G. Personality Predicts Decision Making Only When Information Is Unreliable. Anim Behav [Internet]. 2013;86(3):633–9.

4.	Fragaszy DM, Visalberghi E. Social Processes Affecting The Appearance Of Innovative Behaviors In Capuchin Monkeys. Folia Primatol [Internet]. 1990;54(3–4):155–65.

5.	Regan BC, Julliot C, Simmen B, Viénot F, Charles-Dominique P, Mollon JD. Fruits, Foliage And The Evolution Of Primate Colour Vision. Philos Trans R Soc Lond B Biol Sci [Internet]. 2001 Mar 29 [cited 2014 Oct 27];356(1407):229–83.

6.	Noser R, Byrne RW. Travel Routes And Planning Of Visits To Out-Of-Sight Resources In Wild Chacma Baboons, Papio Ursinus. Anim Behav [Internet]. 2007 Feb [cited 2014 Oct 23];73(2):257–66.
