# Supplementary material for: Exploring individual variation in associative learning abilities through an operant conditioning task in wild baboons
Source: PLoS One. 2020 Apr 6;15(4):e0230810. doi: 10.1371/journal.pone.0230810 (PMC7135308; doi:10.1371/journal.pone.0230810)
Supplement: Attachment — Submitted filename: Response to reviewers (final).docx [file pone.0230810.s007.docx]

**Response to reviewers**

**We would like to thank the comments of the Academic Editor and two anonymous reviewers for their helpful comments and suggestions. Our responses below are presented on a point-by-point basis in blue and bold. When relevant, we include the line numbers that correspond to changes made in the manuscript version without track changes.**

1. L 40: two full stops (typo)

**This has been corrected.**

2. L72-73: the sentence is a bit unclear, I suggest to modify it as follows: “Conversely, the failure of captive animals to solve tasks that they would never encounter in the wild may equally distort our estimate of that species’ cognitive abilities”.

**This sentence has been modified as suggested.**

3. L 83: two square brackets (typo)

**The additional bracket has been removed.**

4. L 135: I suggest to delete the word “personality” here, since personality is a complex construct; similarly, on l 480 I suggest to replace “personality” with “neophilia”

**All mentions of “personality” have been replaced by the work “neophilia”.**

5. L 323: the parenthesis at the end of the sentence is missing

**This has been added.**

6. L 422, 540 please replace the semicolon with a comma

**Done.**

7. L 544 (and 561) since “tendency” has a very precise meaning in statistics (0.05 < p < 0.1), unless this is the case, I suggest to replace this word with an alternative one.

**We have modified each sentence to replace the word “tendency”. In L 544-545 the sentence now reads: “This interpretation is supported by our observation that the baboons left slightly more bitter kernels uneaten”. In L562-563 the sentence now reads: “Such a result may be indicative of a species’ selective attention towards red food items”.**

8. L 551 please erase the comma after “study”

**Done.**
